# Telehealth for children and adolescents with chronic pulmonary disease: systematic review

**DOI:** 10.1590/1984-0462/2024/42/2022111

**Published:** 2023-05-15

**Authors:** Adriana Virgínia Barros Faiçal, Laís Ribeiro Mota, Danilo d’ Afonseca Correia, Larissa Prazeres Monteiro, Edna Lúcia de Souza, Regina Terse-Ramos

**Affiliations:** a Universidade Federal da Bahia, Salvador, BA, Brazil.; b Casa Hunter, São Paulo, SP, Brazil.; c Hospital Martagão Gesteira, Salvador, BA, Brazil.

**Keywords:** Telehealth, Asthma, Cystic fibrosis, Quality of life, Telessaúde, Asma, Fibrose cística, Qualidade de vida

## Abstract

**Objective:**

To revise the impact of telehealth on the quality of life, reduction in pulmonary exacerbations, number of days using antibiotics, adherence to treatment, pulmonary function, emergency visits, hospitalizations, and the nutritional status of individuals with asthma and cystic fibrosis.

**Data source:**

Four databases were used, MEDLINE, LILACS, Web of Science and Cochrane, as well as manual searches in English, Portuguese and Spanish. Randomized clinical trials, published between January 2010 and December 2020, with participants aged 0 to 20 years, were included.

**Data synthesis:**

Seventy-one records were identified after the removal of duplicates; however, twelve trials were eligible for synthesis. Included trials utilized: mobile phone applications (n=5), web platforms (n= 4), mobile telemedicine unit (n=1), software with an electronic record (n=1), remote spirometer (n=1), and active video games platform (n=1). Three trials used two tools, including telephone calls. Among the different types of interventions, improvement in adherence, quality of life, and physiologic variables were observed for mobile application interventions and game platforms compared to usual care. Visits to the emergency department, unscheduled medical appointments, and hospitalizations were not reduced. There was considerable heterogeneity among studies.

**Conclusions:**

The findings suggest that better control of symptoms, quality of life, and adherence to treatment can be attributed to the technological interventions used. Nevertheless, further research is needed to compare telehealth with face-to-face care and to indicate the most effective tools in the routine care of children with chronic lung diseases.

## RESUMO

**Objetivo:**

Revisar o impacto da telessaúde na qualidade de vida, redução das exacerbações pulmonares, número de dias em uso de antibióticos, adesão ao tratamento, função pulmonar, visitas à emergência, hospitalizações e estado nutricional de indivíduos com asma e fibrose cística.

**Fontes de dados:**

Foram utilizadas quatro base de dados, sendo, MEDLINE, LILACS, *Web of Science* e Cochrane, além de pesquisas manuais nos idiomas inglês, português e espanhol. Foram incluídos ensaios clínicos randomizados, publicados no período de janeiro de 2010 a dezembro de 2020, com participantes de 0 a 20 anos.

**Síntese dos dados:**

Setenta e um registros foram identificados após a remoção das duplicatas e doze estudos foram elegíveis para síntese. Os ensaios utilizaram aplicativos para celular (n=5), plataformas da *web* (n= 4), unidade de telemedicina móvel (n=1), *software* com registro eletrônico (n=1), espirômetro remoto (n=1) e plataforma ativa de *videogames* (n=1). Três ensaios utilizaram duas ferramentas, incluindo chamadas telefônicas. Entre os diferentes tipos de intervenções, observou-se melhora na adesão, qualidade de vida e de variáveis fisiológicas para intervenções de aplicativos móveis e plataformas de jogos em comparação com os cuidados habituais. Visitas ao pronto-socorro, consultas médicas não agendadas e internações não foram reduzidas. Houve considerável heterogeneidade entre os estudos.

**Conclusões:**

Os achados sugerem que a melhora do controle dos sintomas, da qualidade de vida e da adesão ao tratamento podem ser atribuídos às intervenções tecnológicas utilizadas. No entanto, mais pesquisas são necessárias para comparar a telessaúde com o atendimento presencial e indicar as ferramentas mais efetivas na rotina de cuidados à população infantil com doenças crônicas pulmonares.

## INTRODUCTION

Chronic childhood diseases affect the body’s functions, and the child’s activity and participation levels, interfering with the well-being and quality of life.^1^ In the face of lung diseases, the management of children and adolescents with bronchial asthma and cystic fibrosis (CF) requires a multidisciplinary approach with a focus on symptom control and treatment of comorbidities, encouraging adherence, and promoting health education.^2,3,4^ Thus, among the tools available for the provision of care, telehealth has proved to be an opportunity to deliver healthcare from distance, promoting the democratization of care and access to specialized services.^5,6,7^

Telehealth can include telemonitoring and telerehabilitation, and the interventions include tools such as websites, smartphone applications (apps), text message reminders, store-and-forward, remote monitoring of symptoms, and videoconference. The activities can be synchronous or asynchronous.^8^ Many publications demonstrated positive effects of telehealth, not only on the quality of life,^9^ physical activity improvement^10^, adherence to treatment,^11^ and controlling exacerbations of chronic diseases,^8^ but also on reduced costs^12^ and greater satisfaction of patients.^13^

We aimed to systematically review the literature to:

1. Determine the impact of telehealth outpatient care on the quality of life of children and adolescents with asthma and cystic fibrosis,
2. Assess the reduction in exacerbations of pulmonary symptoms and the number of days using antibiotics,
3. Check adherence to the treatment,
4. Identify the impact of pulmonary function tests and nutritional status, and
5. Assess the number of emergency visits, and hospitalizations post-intervention.

## METHOD

The systematic review is registered and the protocol is available in the International Prospective Register of Systematic Review (PROSPERO) database, under number CRD42021219892. We followed the procedures described in the protocol recommendations for the publication of systematic reviews Preferred Reporting Items for Systematic Review and Meta-Analysis Protocols (PRISMA-P).^14^

The search strategy, inclusion criteria, exclusion criteria, and analysis plan were specified in advance and are documented in the protocol. Table 1 summarizes the PICOS (Population, Intervention, Comparison, Outcome, and Setting) strategy. We searched four databases — MEDLINE, LILACS, Web of Science and Cochrane — and did manual searches in English, Portuguese, and Spanish. We included randomized controlled trials, published from January 2010 to December 2020, with participants aged 0 to 20 years. The Search terms were asthma OR cystic fibrosis OR telehealth terms such as telemonitoring, telecare, telehomecare, telephone monitoring, telemedicine, home web-based intervention, telerehabilitation, limited to clinical trials.

**Table 1.** Search strategy – Inclusion and exclusion criteria, data range, and sources of searches.

**Population**	Children, adolescents, and young adults (0–20 years old) with a clinical diagnosis of severe asthma or cystic fibrosis.
	We excluded individuals with greater impairment of lung function and associated heart disease
**Intervention**	Any telehealth intervention with any currently available device, such as a smartphone, tablet, smart TV, or computer.
	We did not include hybrid interventions
**Comparator**	Individuals who were not provided with or did not have access to telehealth and face-to-face healthcare
**Outcomes**	Health-related quality of life, adherence to treatment, pulmonary function, pulmonary disease exacerbation, hospitalization, nutritional status, emergency visit, antibiotic therapy uses
**Settings**	Any health care setting
**Study design**	Studies were included if they were randomized controlled trials (RCTs)
**Date range**	The date range for all searches was January 1, 2010, to December 31, 2020
**Databases**	MEDLINE, LILACS, Web of Science and Cochrane library

Initial screening for articles was performed by two independent reviewers (authors AVBF and DFC) based on titles, abstracts, and keywords. It was verified whether the selected articles met the inclusion criteria (see at Table 1, the PICOS description). Papers with no abstract available were added to a full-text review. We categorized articles into eligible, not eligible, and uncertain. To confirm eligibility, papers with no available abstract and those that fell into the uncertain category were read in full text independently. Both reviewers resolved any disagreement through discussion or, if required, through consultation with another review author (LRM). The reviewers, thereafter, discussed which articles would be included in the final review and reached a consensus.

A data collection form was used to obtain information. Study characteristics extracted encompassed the author’s name, publication year, country of the study, method, demographics of participants, clinic diagnostic, sample size, intervention duration, type of intervention, and control setting, features of the telehealth, and clinical outcomes.

Two reviewers (DFC and AVBF) assessed and documented the methodological quality of included clinical trials using the Jadad scale (Table 2).^15–27^ The basic Jadad score is determined based on five questions which answers are “yes” (1 point) or “no” (0 point). There are no in-between marks. The questions are: “Was the study described as random?”, “Was the randomization scheme described and appropriate?”, “Was the study described as double-blind?”, “Was the method of double blinding appropriate? (Were both the patient and the assessor appropriately blinded?)”, and “Was there a description of dropouts and withdrawals?”. Thus, the questions are summarized in three domains: randomization, double blinding, and withdrawals/dropouts. The range of the score quality is 0–2 Low, or 3–5 High.

**Table 2.** Risk of bias across interventions.

Author	Randomization	Blinding	Patients (n)	Total	Range of score quality
Deschildre et al.^18^	0	0	1	1	Low
Bender et al.^19^	0	0	1	1	Low
Montalbano et al.^17^	2	0	1	3	High
Stukus et al.^20^	0	0	1	1	Low
van den Wijngaart et al.^27^	2	0	1	3	High
Perry et al.^21^	0	0	1	1	Low
Halterman et al.^22^	2	1	1	4	High
Del Corral et al.^16^	2	0	1	3	High
Kosse et al.^26^	2	0	1	3	High
Stark et al.^23^	2	0	1	3	High
Gustafson et al.^24^	2	2	1	5	High
Perry et al.^25^	0	0	1	1	Low

A meta-analysis was precluded due to heterogeneity of telehealth interventions, intervention intensity, face-to-face comparators, and study duration. We presented results narratively, with tables for illustration.

The results of the data synthesis were discussed by the multidisciplinary team comprising the authors of this review, who have expertise in telehealth and the management of patients with asthma and cystic fibrosis.

## RESULTS

**Figure 1.**
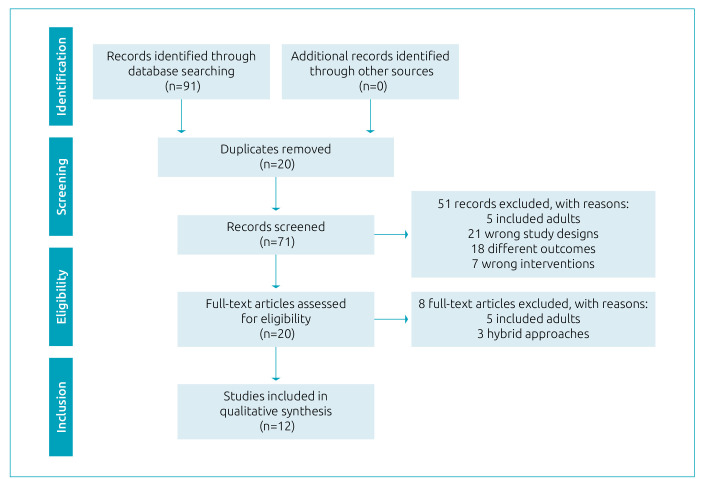
Study identification and selection process. The flow of information through the different stages of the systematic review according to the Preferred Reporting Items for Systematic Review and Meta-Analysis Protocols guidelines.

The identified papers, the screening process, and the final number of studies included are detailed in the PRISMA flowchart (Figure 1). In summary, out of 91 papers, 12 were finally included.

The detailed characteristics of included studies are summarized in Tables 3 ^19–25^, and 4.^16–19,26,27^ The 12 interventions were conducted from 2012 to 2019 across the world: one of each in Spain,^16^ Italy,^17^ and France,^18^ seven in the USA,^19-25,^ and two in the Netherlands.^26,27^ The studies are all randomized controlled trials (RCTs), including one cluster RCT and two pilot trials. The risk of bias across interventions is summarized in Table 2.

**Table 3.** Clinical outcomes of the included interventions; publications from the United States of America.

Author/design	Participants (n), age range (years), diagnosis	Intervention	Outcomes	
Gustafson et al.^24^/RCT	n=301 Parents or legal responsible who had children aged 4–12 years with asthma	CHESS+CM	SFD improved for the CHESS+CM group (OR 1.38; p=0.010) and less for the control group (OR 1.20; p=0.29). There were no between-group differences (OR 0.18; p=1.000). ACQ improved significantly for CHESS+CM and not significantly for the control group (OR -0.11; p=0.220).	
Bender et al,^19^/Pragmatic RCT	n=871 3–12 years old Asthma	Health Care Technologies	Adherence in the IG was 25.4% higher than that in the UC group. No between-group differences for hospitalizations, ED visits, and β2-agonist use.	
Stark et al.^23^/Pilot randomized trial	n=20 Mothers of children with CF ages 4–9 years	Web intervention BIC	Children in BIC gained an average of 116% of expected daily weight and UC gained an average of 52% of the expected daily weight, and increased by 27% from their baseline EER.	
Perry et al.^25^/Randomized pilot study	n=34 12–17 years old Asthma	Personalized smartphone-based AAP	There was change in ACT for the smartphone group (p=0 .040) but not for the paper group (p=0.640).	
Halterman et al.^22^/RCT	n=400 3–10 years old Asthma	SB-TEAM	SB-TEAM group had more SFDs compared with eUC group (11.6 vs 10.97; difference, 0.69; 95%CI 0.15–1.22; p=0.010), fewer symptom nights, and days with limited activity. SB-TEAM group had fewer ED visits or hospitalizations (7 vs 15%; OR 0.52; 95%CI 0.32–0.84).	
Perry et al.,^21^/Cluster RCT	n=393 7–14 years old Asthma	School-based telemedicine intervention	Family activity domain of the CHSA, improved for the UC group, but not the IG (p=0.020). The use of peak flow to monitor asthma was 45% UC group vs 79% IG p<0.0001, there was no change in AMR, PedsQL did not reach statistical significance (p=0.060)	
Stukus et al.,^20^/Prospective RCT	n=200 6 months–21 years old Asthma	AC	AC participants had a 72% decrease in total number (29 vs 11; mean 0.3 vs 0.11; p=0.020) of UC visits; controls had no significant difference (25 vs 19; 0.26 vs 0.2; p=0.430). AC participants reported improvement in asthma management 6 months after study enrollment (79 vs 64%; p=0.060)

RCT: Randomized controlled trial; CHESS: Comprehensive Health Enhancement Support System; CM: a monthly telephone call to the parent from an asthma nurse case manager; SFD: symptom free days; OR: odds ratio; ACQ: Asthma Control Questionnaire; IG; intervention group; UC: usual care; ED: Emergency Department; CF: cystic fibrosis; BIC: BeInCharge.org; EER: estimated energy requirement; AAP: asthma action plan; ACT: childhood asthma control test; SB-TEAM: School-Based Telemedicine Enhanced Asthma Management; eUC: Enhanced usual care; CHSA: Children’s Health Survey for Asthma; AMR: asthma medication ratio; PedsQL: Pediatric Quality of Life; AC: asthma care.

**Table 4.** Clinical outcomes of the included interventions; publications from European countries.

Author, country, design	Participants (n), age range (years), diagnosis	Intervention	Outcomes
Deschildre et al.,^18^ France, prospective RCT	n=44 6–16 years, asthma	Daily home telemonitoring	PAQLQ scores within each of the two groups (median in the HM group was -0.3 (-4.2–1.1) (p=0.240) and in the CT group was -0.1 (-3.8–0.5) (p=0.150).
Del Corral et al.,^16^ Spain, single-blinded RCT	n=40 7–18 years, cystic fibrosis	Home-based AVG Programme	The change in scores showed significant differences for the 6MWT and MSWT compared to the CT. All muscle strength variables increased after the intervention, as did HRQoL.
Kosse et al.,^26^ Netherland, cluster RCT	n=261 12–18 years, asthma	ADAPT	Total app use was not associated with a difference in self-reported adherence (p=0.120). CARAT (p=0.260), adherence questions (p=0.650), short movies p=0.800) and peer chat (p=0.210). Logged activity in pharmacist chat positively affected self-reported adherence (p=0.030)
Montalbano et al.,^17^ Italy, non-blinded RCT	n=50 6–11 years, asthma	MyTEP	PAQLQ score increased in both groups. Increase in C-ACT score was only found in MyTEP. No differences were found in the MARS-9 score. Lab spirometry and BD response (%) values were similar
van den Wijngaart et al.,^27^ Netherland, Prospective multicenter unblinded RCT	n=210 6–16 years, asthma	VAC	Number of SFD, after 16 months, was in favor of the VAC compared with UC (difference of 1.23 days; 95%CI 0.42–2.04; p=0.003). For the C-ACT, there was a difference in the mean outcome of 1.17 points (95%CI 0.09–2.25; p=0.030) in favor of VAC. It was demonstrated at 16 months compared with UC, in children aged 6–11 years. In children aged 12–16 years, asthma control was similar after 16 months of follow-up, as the ACT score did not differ between both groups (0.88 points; 95%CI -0.41–2.16; p=0.180)

RCT: Randomized controlled trial; PAQLQ: Pediatric Asthma Quality-of-Life questionnaire HM: home monitoring; CT: conventional treatment; AVG: Active Video Game; 6MWD: 6-min walk test; IG: Intervention Group; MSWT: modified shuttle walk test; HRQoL: health-related quality of life; ADAPT: Adolescent Adherence Patient Tool intervention; CARAT: Control of Allergic Rhinitis and Asthma Test; MyTEP: my therapeutic education program; C-ACT: Childhood Asthma Control Test; MARS-9: Medication Adherence Report Scale; BD: bronchodilator; SFD: symptom free days; VAC: virtual asthma clinic; UC: usual care; 95%CI: 95% confidence interval.

Overall, 58% of the reviews were graded as high quality, while the remaining 42% were of low to critically-low quality. The most common critical weakness in clinical trials was the failure to include or clearly explain the randomization and blinding. Most trials, however, performed adequate methodological procedures and used satisfactory techniques to test their hypothesis. Most reviews adequately followed PICO elements.

The number of participants for each study ranged from 20 to 871, and individuals were recruited from pediatrics hospitals, health organizations, outpatient clinics, tertiary referral centers, and associations. The studies included an age range from 6 months to 21 years, 1 trial included children under 3 years,^20^ and 3 studies involved caregivers or parents.^21,23,24^ In this review, 2 studies were conducted in CF patients and 9 in asthmatics. The duration of interventions ranged from 1 to 24 months.

For the 12 telehealth interventions, the following tools were applyed: five mobile phone apps,^17,20,23,25,26^ one of each web-based portal,^27^ mobile telemedicine unit,^22^ telemedicine (live interactive video) and telephone,^21^ e-Health program and telephone,^24^ software with an electronic record,^19^ and two studies used devices such as spirometer remote^18^ and active video games.^16^

In most studies, the comparator was the participants who received usual care. However, two studies used the mobile — Health Program (m-Health) and paper-based app — for the control group.^17,25^

Outcomes are summarized in Tables 3 and 4. The positive effect on asthma control and more symptom-free days compared to usual care were reported in four studies.^17,22,25,27^ There were no statistically significant differences between groups in the two studies.^21,24^

### Quality of life

One study demonstrated improvement in quality of life in asthmatic participants, although there was no change in spirometry^17^. Additionally, one study involving people with CF identified increasing health-related quality of life (HRQoL), such as improving in 6-min walk test (6MWT), modified shuttle walk test (MSWT), and muscle strength.^16^ Finally, two studies reported that there were no significant changes in scores after intervention.^18,21^

### Exacerbation

Only two studies mentioned exacerbation as an outcome. A treatment based on daily forced expiratory volume in the first second (FEV1) monitoring, with medical feedback, did not reduce asthma exacerbation, besides, the use of a virtual asthma clinic through a web-based portal demonstrated no differences between groups.^18,27^

### Adherence

Most of the studies did not report adherence in their outcomes. The study that used speech recognition software and electronic health records demonstrated more adherence in the intervention group,^19^ and the study that used the Home-Based Active Video Game Programme verified that long-term adherence progressively decreased.^16^ One study that used e-Health plus telephone call and app as intervention, presented no difference in adherence between the intervention group and the usual care.^24^

One study that incorporated support for pharmacists improved self-reported adherence when adolescents sent messages to this particular professional. However, adherence did not change when participants used apps, short movies, or peer chats.^26^

### Nutrition

Only one study evaluated this outcome. Despite the small sample, the authors reported daily weight gaining and increasing estimated energy requirement in comparison to the control group.^23^

### Hospitalizations and emergency department visits

Within the 12 studies, only one reported that children in the intervention group had lower hospitalization and emergency department (ED*)* visits rates than the control group (7% vs 15%; odds ratio 0.52; 95%CI 0.32–0.84).^22^ No difference was demonstrated in three studies that used different tools to provide telehealth.^19,20,27^ In this review, the other studies did not mention hospitalization and ED as an outcome.

## DISCUSSION

Most of the interventions included in this review demonstrated positive results. However, multiple features such as app, web-based portal, home-based game, and mobile telemedicine unit were used, and outcomes were variable. Our findings suggest that telehealth could be an opportunity to improve clinical outcomes such as symptoms control, engagement, and adherence. Moreover, only one study reported change in the number of hospitalizations and emergency visits, quality of life of parents or caregivers, and satisfaction with the use of telehealth.^22^

Despite the many tools available to provide remote healthcare, results are heterogeneous. Some trials with negative outcomes presented reduced sample size and short-term exposure to the approach, and these aspects could influence their findings. Additionally, the outcomes evaluated were also different. For example, exacerbation is an important health-related outcome and only two studies reported this.^18,27^

Participants’ follow-up was not mentioned in many different trials. Nonetheless, just one study that performed follow up verified that the long-term adherence decreased, and fewer outcomes related to health were kept.^16^ Physiological variables such as increasing muscle strength, cardiorespiratory performance, and weight gain were explored in two studies and presented positive effects of the intervention.^16,23^ In contrast, pulmonary function did not differ in these three studies just mentioned.^17,18,27^

According to this systematic review protocol, the research was conducted from February 2021 to December 2021 and included research published between 2010 and 2020. Two studies were published after 2020,^28,29^ and both showed positive outcomes in quality of life and functional status in children diagnosed with CF, as well as in better asthma control, fewer ED visits, and hospitalizations. Otherwise, Bitar and Alismail presented in their recent systematic review of the role of eHealth, telehealth, and telemedicine that despite the large body of literature published today, there is a lack of evidence for studies, suggesting the need for more rigorous methodology.^30^

Another systematic review that examined the effectiveness of mobile-health (mHealth) apps for pediatric chronic disease management described positive outcomes, including improved adherence and reduced exacerbations in children with asthma. However, the authors emphasized that many studies have limitations related to ethical and privacy issues, as well as the necessity to improve the methods used.^31^

Our systematic review provides an evidence-based primary study that used different tools to perform telehealth. The focus of the review was to evaluate the main outcomes of clinical relevance on the healthcare of people diagnosed with asthma and CF. However, in the face of different methodologies and resources used, the results were variable. A further limitation arises from the scarcity of publications available during the period of this study that included telehealth for the treatment of pediatric patients with chronic pulmonary disease.

In addition, the results found could have differences compared to new studies in progress. Prior to the COVID-9 pandemic, patients using telehealth were a self-selected audience with appropriate characteristics, for whom healthcare providers would prioritize in-person appointments in cases of increased risk or medically complex conditions. Post-March 2020, the growth of telehealth resulted in many resources and platforms available for individuals’ practices worldwide.

In terms of implications for clinical care and future research, treatment options for complex chronic diseases are often far away and difficult to access.^32,33^ Therefore, telehealth could have the potential to increase access to services where appropriate healthcare structures and offers are missing, as it promises to increase patient access, improve the quality of services, and reduce costs in healthcare.^8,34,35^ Despite this, other aspects require attention because different experiences have been reported among lower income people, reduced literacy, and racial minorities. The challenges are associated with home conditions, broadband infrastructure, and access to technology in general.^36,37^

In the digital era, many features are available such as applications, e-Health, web portal, platform, and telemedicine mobile.^8^ However, there is insufficient evidence to identify better tools that encourage patients to use in their care routine. The approaches must be personalized and tailored to the patients’ needs and preferences, as well as the assessment of parents’ satisfaction. Additionally, a multidisciplinary team must be trained, in order to acquire competencies on legal aspects and best practices in communication.^37^

Telehealth has been expanding worldwide and has proven to be a great opportunity to deliver effective healthcare, despite requiring robust studies that establish the long-term results.^38,39^ Although, this model of care involves a new paradigm to access health services with advantages for patients, parents, professionals, and institutions, the face-to-face model must not be neglected. Some authors suggest a hybrid system, considering the adequate decision about eligible patients and their respective demands.^35^

Hence, the development of researches that evaluate the efficacy of telehealth, specific devices associated with improved outcomes, and better strategies for equalizing access to telehealth would be essential to bring important contributions to enhancing the system of healthcare.

Telehealth is considered a strategy to support healthcare through information and communication technologies and has been used for various health conditions and age groups. Numerous publications are available and emerging rapidly, but there are few studies involving pediatric patients with chronic lung disease.

This is a broad model of care with many opportunities, but also challenges. There is no single approach, and different resources can be used.

In conclusion, the results of this review suggest that these interventions lead to improved symptom control, quality of life, and treatment adherence in both asthmatic and CF patients. Nevertheless, further robust research is needed to compare telehealth with face-to-face care and to indicate the most effective tools in the routine care of children with chronic lung diseases.
